# Ecologically Relevant Decisions and Personality Configurations: A Theoretical–Clinical Proposal Considering Quantum Cognition

**DOI:** 10.3390/brainsci15121300

**Published:** 2025-12-01

**Authors:** Raffaele Sperandeo, Lucia Luciana Mosca, Chiara Scognamiglio, Valeria Cioffi, Enrico Moretto, Natascia De Lucia, Benedetta Muzii, Nelson Mauro Maldonato

**Affiliations:** 1 Department of Neurosciences and Reproductive and Odontostomatological Sciences, University of Naples Federico II, 80131 Naples, Italy; raffaele.sperandeo@gmail.com (R.S.);; 2 Sipgi, Post Graduate School of Gestalt Psychotherapy, Torre Annunziata, 80058 Naples, Italy

**Keywords:** decision-making process, quantum cognition, personality, brain

## Abstract

A situational individual’s mental state contains the dynamic interaction between explicit, conscious cognitive processes and implicit automatisms that develop outside of consciousness. The influence of implicit and unaware processes, often underestimated, manifests itself in the speed with which we process information, in immediate emotional responses, and in the emergence of intentions even before they are accessible to awareness. The brain activity that precedes a decision manifests itself several milliseconds before the subject reports the conscious intention to act. This phenomenon highlights how awareness emerges after the decision-making process. The Quantum Cognition model proposes an alternative theoretical framework to classical logic to explain complex cognitive phenomena such as ambivalence, overlapping intentions, and sudden changes in perspective. The aim of this paper is to propose the QC model as a powerful formal strategy for describing the mind as a dynamic, probabilistic, and context-sensitive system.

## 1. Introduction

A situational individual’s mental state contains the dynamic interaction between explicit, conscious cognitive processes and implicit automatisms that develop outside of consciousness. Neuroscientific and psychological literature has extensively documented the interaction between these two systems: one explicit, deliberative, and conscious, and one implicit, automatic, and not conscious [1,2].

The influence of implicit and unaware processes, often underestimated, manifests itself in the speed with which we process information, in immediate emotional responses, and in the emergence of intentions even before they are accessible to awareness [3]. Functional neuroimaging studies have shown that many decisions are initiated unconsciously at the neural level, anticipating the subject’s awareness by several hundred milliseconds [4,5].

Furthermore, computational models and experiments in the field of implicit psychology emphasize the decisive role of these components in shaping preferences, moral judgments, and social behaviors, even in the absence of conscious deliberation [6,7]. In ecologically valid decision-making processes (such as those that occur in emergency situations, social interactions, or moral conflicts), the automatic, affective, and pre-reflective component often plays a dominant role [8,9,10].

As clarified by the pioneering work of Libet [11], the brain activity that precedes a decision—the so-called readiness potential—manifests itself several milliseconds before the subject reports the conscious intention to act. This phenomenon has been confirmed and expanded upon by studies using functional magnetic resonance imaging and electroencephalography techniques [12,13], highlighting how awareness emerges after the decision-making process.

These results support the hypothesis that awareness does not play a direct causal role in determining action, but rather a narrative, integrative, and justificatory function of subjective experience [14,15]. From this perspective, it would act by constructing temporal and motivational coherence between internal and external events, rather than as a primary generator of behavioural choices. This does not imply a denial of free will but requires its reformulation in terms of distributed and multilevel processes, which include bodily, affective, and relational dimensions [16,17].

This is where the innovative contribution of the Quantum Cognition (QC) model comes in, proposing an alternative theoretical framework to classical logic to explain complex cognitive phenomena such as ambivalence, overlapping intentions, and sudden changes in perspective [18]. In summary, the QC model offers a powerful formal strategy for describing the mind as a dynamic, probabilistic, and context-sensitive system [19], opening up new theoretical possibilities for integrating preverbal and affective dimensions into the heart of the decision-making process. QC ultimately offers a methodological tool for modeling the behavior and symptomatology of subjects with personality disorders, integrating it into a phenomenological view of personality and psychopathology. This theory offers the possibility of organizing theoretical prediction models consistent with clinical experience, without theorizing a quantum structure of the brain.

The article proposes a theoretical model and its clinical implications, without presenting experimental data, and aims to offer an innovative interpretative framework for complex psychic phenomena that are difficult to model using classical paradigms.

The equations of quantum theory, applied to psychic phenomena according to the recent QC paradigm, allow us to formally represent mental dynamics characterized by ambivalence, decision fluctuations, sensitivity to context, and unconscious behaviors. These characteristics, typical of psychic functions, are very evident in some pathological forms such as personality disorders or clinical conditions characterized by emotional instability.

The perspective described in the article is relevant for the development of empirical studies, as it provides the tools to formalize concepts that are currently difficult to describe, such as the overlap of emotional states, the interference of relational dynamics, or the affective-relational tipping point, in experimental designs, assessment protocols, and calculation procedures [20].

Furthermore, the model offers a coherent theoretical basis for constructing clinical tools that take into account subjective variability, the role of lived experience, and temporal and relational dimensions, and it stimulates the development of transdisciplinary research between psychopathology and computational sciences.

In terms of research methodology, the first part of the article presents a theoretical discussion, without mathematical formalism, of an analytical model capable of overcoming the existing gap between classical calculation methods applied to decision studies (in particular dual process or Bayesian models), which are unable to describe the chaotic and complex aspects that characterize real psychic phenomena.

In the second part, the paper proposes the application of this model to studies on personality disorders that show the non-linear elements of mental life in an extreme way.

More specifically, dual-process models describe human decisions as the result of two systems: an intuitive, rapid, and automatic (unconscious) system and a deliberative, slow, and rational (conscious) system. Bayesian models describe decision-making as a consistent updating of subjective probability based on new evidence. Both models assume a stable determination of the subject’s mental state, where preferences and intentions are always well defined and distinct.

In the real world (especially in clinical settings), these assumptions are continuously violated. For example, a borderline patient may simultaneously desire and fear the closeness of a significant figure. This mental state is not a linear combination of two opposing intentions, but a genuine coexistence of incompatible intentions, which cannot be described by a classical probability distribution or a logical sequence based on the dualism of unconsciousness/consciousness.

In these situations, decisions and behaviors emerge from a potential configuration of the mental state, which stabilizes and defines itself only at the moment of action or verbalization. This is the concept of the “collapse mental state” described by Quantum Cognition. The QC model, therefore, enables a formal representation of the non-linear coexistence of mental states and the potential nature of choices, thus filling a methodological gap that classical models do not address.

This perspective is particularly suited to understanding personality psychopathology, where instability of the self and discontinuity of decision-making processes are central characteristics. The QC-based approach to personality and its disorders (which is an approach currently mainly used to study human decisions) is at the forefront of this model, which draws on the mathematical formalisms of quantum mechanics.

### Structure of the Article

This work is a theoretical-conceptual proposal based on the integration of Quantum Cognition (QC) formalism, psychological models of decision-making, and personality psychopathology. The aim is not to present empirical results, but to construct a coherent and formally structured theoretical framework capable of representing complex and non-linear psychic phenomena such as ambivalence, instability, impulsive behavior, and context dependence, which are not adequately modeled by classical theories.

The methodology followed is divided into three levels:

1. Critical review of existing models. The epistemological and descriptive limitations of traditional decision-making models were analyzed, highlighting their inconsistencies with observable clinical phenomena.
2. Conceptual formalization inspired by QC. The fundamental concepts of quantum formalism (superposition, interference, state collapse, non-commutativity, Hilbert space) were introduced and described, translating them into psychological and clinical constructs.
3. Clinical exemplification and operational proposals. The model is applied to phenomena observable in clinical practice, particularly in subjects with a borderline personality organization, proposing hypothetical scenarios and concrete experimental protocols (including both controlled clinical studies and computational simulations). This clarifies the operationalization of QC concepts for clinical studies.

Through this structure, the work aims to propose a theoretically and epistemologically grounded model that is clinically relevant and methodologically explorable. It is conceived as a basis for the development of assessment tools and therapeutic interventions that are more sensitive to the complexity of subjective experience in the psychopathological field.

## 2. Decision-Making Between Automatism and Awareness

Decisions, in their many forms, arise from a logical sequence of rational evaluations. Although classical decision models, such as Expected Utility Theory [21], have proposed a normative approach based on formal rationality, research in cognitive psychology and behavioral sciences has consistently shown that human choices systematically diverge from these ideal models. Studies on decision-making processes in natural and real-world contexts have shown that humans rely on a varied repertoire of cognitive heuristics and shortcuts, often activated implicitly and unintentionally [22,23].

These decision-making strategies, which are developed to ensure speed and efficiency in environments marked by uncertainty, time constraints, and incomplete information, are generally functional, though not free of systematic biases [24]. Heuristics, such as availability, anchoring, or representativeness, reduce the complexity of the decision-making task but introduce problematic deviations from formal and statistical logic [25]. However, more recent studies in the field of ecological rationality have re-evaluated the effectiveness of some of these strategies, showing that in everyday contexts, heuristics can be more useful and effective than more computationally complex approaches [26,27].

The cognitive continuum model, introduced by Hechtlinger [28], suggests that human thinking unfolds along an axis that oscillates between intuitive, rapid, automatic modes and analytical, slow, deliberative modes. This oscillation depends largely on the characteristics of the task, the level of uncertainty, and time pressure. Decisions made under conditions of ambiguity or urgency tend to favor intuitive processes, whereas those that require formal justification or complex evaluation activate more analytical modes [29,30].

Laura Quante’s SRK (Skills-Rules-Knowledge) model [31] complements this perspective and proposes a hierarchy of cognitive processing levels in complex environments, such as industrial or critical settings. At the lowest level, behavior is governed by skill-based automated abilities, as seen in highly trained experts. The rule-based intermediate level involves the application of learned rules to known situations. Finally, the highest, knowledge-based level requires explicit inferences, problem solving, and creative reasoning in new or unexpected situations [32]. This hierarchy reflects the progressively greater demand for cognitive resources and the increasing involvement of awareness in the control of action.

The Recognition-Primed Decision (RPD) model, developed by Gary Klein [33], fits into this framework by showing how, in high-pressure contexts, effective decision-making emerges from the immediate recognition of prototypical situations and the activation of experiential mental schemas. According to this model, the expert decision-maker does not actively compare alternatives; rather, the expert quickly recognizes a familiar situation and mentally simulates its consequences to verify its plausibility. If the simulation aligns with the objective, the action is carried out; if not, an iterative process of adaptation is activated. This model has been widely applied in fields such as aviation, emergency medicine, military leadership, and crisis management [34,35].

Overall, these models provide a useful theoretical framework for understanding the plasticity of human thought, which adapts to environmental demands by modulating the degree of automatism, deliberation, and simulation involved in decision-making. The result is a complex and layered picture of human decision-making, which cannot be understood solely in terms of rational calculation but must also integrate affective, bodily, contextual, and pre-reflective dimensions.

### 2.1. Decision-Making Styles, Emotional Pressure, and Personality

Decisions made under emotional and time pressure are quite vulnerable to cognitive distortions that can lead to performance failures. This is particularly evident in high-stakes contexts—such as sports performance, emergency situations, or assessment tasks—where the affective component can interfere with the attentional and mnemonic resources necessary for the execution of the task [36]. One of the most widely studied phenomena in this area is “choking under pressure”, the impairment of performance due to excessive physiological arousal accompanied by hyperawareness of one’s own behavior (heightened self-awareness), which disrupts the automatisms acquired through practice [37,38]. Under these conditions, attention shifts from the external task to internal monitoring (“self-focus”), thereby compromising the efficiency of already established motor and cognitive routines.

The Yerkes-Dodson law [39] describes this dynamic through an inverted U-shaped function, in which performance improves with physiological activation up to an optimal level, beyond which a deterioration in performance is observed due to cognitive overload and emotional tension. This curve, widely confirmed in experimental and applied settings, highlights the need for a balance between activation and control in order to maintain optimal performance [40].

In this context, personality plays a crucial role. Individuals with high levels of neuroticism are more prone to experiencing anticipatory anxiety, rumination, insecurity, and hypersensitivity to the judgment of others [41], all factors that amplify the risk of choking [42,43]. Conversely, traits such as extroversion, openness to experience, and emotional stability are associated with greater resilience to stress, improved cognitive flexibility, and a higher capacity for dynamic adaptation, which act as protective factors against performance collapse under pressure [44], which act as protective elements against performance collapse under pressure [45,46].

This evidence suggests that decision-making performance in high-pressure contexts is not only the product of technical skills or rational preparation, but is also strongly modulated by psychophysiological dynamics, temperamental dispositions, and affective regulation strategies [47].

#### 2.1.1. Adaptive Cognitive and Heuristic Strategies

In the field of everyday decisions, individuals tend to use heuristic strategies, i.e., cognitive shortcuts that reduce computational load, speed up choice, and conserve attentional resources [48,49]. Although heuristic strategies introduce a margin of systematic error, they are adaptive in ecologically valid conditions, where information is uncertain or incomplete, and time is limited.

Among the principal strategies, a distinction can be observed between compensatory and non-compensatory strategies. Compensatory strategies, such as the weighted sum model or multi-attribute utility theory [50], involve weighing the pros and cons: an option can be chosen even if it has shortcomings in some attributes, provided that these are compensated for by advantages in others. Non-compensatory strategies, such as the lexicographic rule or elimination-by-aspects [51], exclude alternatives that do not meet certain minimum criteria, even if they excel in other aspects. The latter strategies are faster but also more susceptible to distortion, especially in the presence of rigid cognitive traits or high emotionality.

The choice between these strategies is modulated by multiple factors: individual (such as experience, level of fluid intelligence, metacognitive abilities), contextual (time available, task ambiguity, perceived risk level), and emotional (mood, anxiety, physiological arousal) [52,53]. Time pressure, for example, tends to favor non-compensatory strategies, while the availability of time and information promotes more analytical approaches.

Psychological literature has also identified different decision-making styles, which represent recurring patterns in the ways choices are approached: deliberative (analytical, thoughtful), impulsive (quick, reactive), intuitive (based on affective signals and experiential patterns), reflective (oriented toward self-evaluation and prediction of consequences) [54,55]. These styles should not be conceived as rigid traits, but as dynamic and situational patterns emerging from the interaction between temperamental dispositions, cognitive content, and characteristics of the decision-making environment.

#### 2.1.2. Towards a Complex Theory of Decision-Making and the Theory of Quantum Cognition

Decision-making can only be understood within a complex framework that integrates multiple levels of analysis: neurobiological, cognitive, affective, personological, and phenomenological. Each choice is a multidimensional act, in which sub-personal components (such as automatic neural processes), personal components (such as biographical history and temperamental traits), and transpersonal components (such as the relational or symbolic context) are dynamically intertwined. The traditional dualism between the unconscious and the conscious is gradually being superseded by models that recognize a fluid and interactive continuity between different levels of information processing [56,57]. From this perspective, the unconscious is no longer conceived exclusively as a repository of repressed content or a source of pathological distortion, but as a generative, probabilistic, and predictive domain: an environment in which anticipations, implicit simulations, and not yet verbalized but potentially orienting trajectories of meaning are configured [58,59].

Within this review, quantum cognition theory is proposed as a tool capable of effectively modeling the complex interactive processes that we highlighted in the first part of the work [60].

Quantum cognition does not claim that the brain functions as a physical quantum system, but rather that certain characteristics of cognition—such as the non-commutativity of mental acts, interference between alternatives, the coexistence of superimposed states, and the “collapse” of decision-making at the moment of action—are best described by a formalism inspired by quantum mechanics. In this framework, indecision is not simply a lack of information, but a superimposed mental state in which multiple options coexist until the decision-making act produces a resolution [61].

This formalism also allows us to model the role of context, which in quantum theory is not external and objective, but interactive and constitutive of the mental state. Thus, improvisation, creativity, and the ability to reformulate scenarios—core features in conditions of ambiguity or novelty—are not regarded as deviations from normative rationality, but as superior adaptive forms of decision-making. They emerge from a cognitive framework capable of sustaining ambivalence, tolerating uncertainty, and producing novel configurations [62].

## 3. Quantum Cognition and Non-Classical Models of Decision Making

In recent years, there has been growing interest in the application of quantum mechanics models to cognitive processes, particularly to decision-making in conditions of uncertainty, ambiguity, and conflict. This approach, known as Quantum Cognition, does not imply that the brain operates according to quantum physical principles—such as the behavior of subatomic particles—but rather that formal models of quantum theory can more effectively represent certain non-classical aspects of human thought [60].

Unlike traditional cognitive models, which assume that preferences, beliefs, and intentions are always defined and stable, the quantum cognition model assumes that mental states are dynamic and contextual. In this perspective, the mind does not have a clear position on all available options at any given moment, but is in a superimposed state in which different possibilities coexist until the decision-making act, influenced by context, emotions, and questioning modes, may not emerge from this state. Just as in quantum physics, a particle can be in multiple states simultaneously until it is observed, so too in human cognition, a decision can exist in a potential state until the moment of choice. This approach has proven particularly effective in modeling paradoxical or seemingly irrational cognitive phenomena, such as context-induced opinion shifts (question order effect), fluctuating consistency of preferences, and ambivalence in emotions or moral judgments. Such phenomena challenge the rules of classical logic and Bayesian probability, but find a more natural representation in the language of quantum probability, which allows for interference, order dependence, and states that are not determined a priori [61,63].

In this sense, Quantum Cognition (QC) represents not only a theoretical evolution but also a paradigm shift: from the mind conceived as a computational system to a fluid and plastic organism, capable of holding together uncertainties, ambivalences, and multiple possibilities until the creative act of decision-making.

### 3.1. Principles of Quantum Cognition

Quantum cognition uses the formalism of quantum mechanics, in particular its conceptual structure, to model cognitive processes that escape the rules of classical logic and conventional probability. The goal is not to claim that the brain functions as a quantum physical system, but that mental behavior under certain conditions—such as uncertainty, ambivalence, decision conflict, or contextual influence—exhibits properties analogous to those described by models of quantum physics. This approach has proved particularly useful in explaining non-linear, contradictory, or dynamically unstable cognitive phenomena that classical psychology struggles to represent coherently [60,61].

In concrete terms, cognitive overlap can be modeled, for example, through experimental decision-making tasks that include conflicting or ambivalent options (e.g., moral choices, interpersonal dilemmas, or time-limited tasks with multiple meaningful alternatives). Subjective ambivalence can be measured using self-report instruments, indecision ratings, or internal-conflict scales. Intra-individual variability in responses (reflecting cognitive instability) and context sensitivity can be studied by manipulating the order or framing of alternatives and testing for non-commutativity effects.

The “collapse of cognitive state” can be operationalized as the moment of final choice, detectable through temporal (latency to response), behavioral (post-decision stability), or affective (subjective arousal at the moment of choice) measures.

The psychological translations of quantum theory concepts can also be studied in physiological terms as the critical point at which emotional intensity or contextual pressure causes a sudden resolution of the ambivalent state [64].

To strengthen the connection between psychological and neurophysiological levels, these paradigms can be integrated with measures of interoceptive functioning and autonomic regulation. For example, Heart Rate Variability (HRV) can be measured to explore the subject’s ability to tolerate overlap without collapsing prematurely. Cerebral oxygen consumption (detected via fNIRS, or with BOLD-fMRI techniques) can be used to monitor metabolic engagement during phases of cognitive ambiguity or in the transition to decision-making. Personality functioning scales can be used to describe the “structure of the individual Hilbert space” and the ability to sustain complex, multiple, or ambivalent mental states [65].

In this way, the theoretical architecture offered by the Quantum Cognition model becomes an epistemological scaffolding for constructing multilevel experimental designs capable of connecting subjective psychological phenomena, clinical personality traits, and neurophysiological indicators within a coherent and formalizable framework.

One of the key concepts is superposition: a mental state can contain multiple alternatives simultaneously, without the subject having yet consciously defined a clear preference. For example, when faced with a complex emotional choice (staying in or leaving a relationship), a person may find themselves simultaneously in both intentional states, oscillating between contradictory impulses. This state of coexistence can be represented more effectively with the concept of superposition than with a classic binary view that presupposes a clear choice already made.

Another crucial concept is the non-commutativity of order: in the quantum cognition field, the outcome of a mental process may depend on the order in which information is presented, or questions are asked.

This means that human thought is not always stable or linearly consistent but can be profoundly influenced by the sequence of stimuli. This effect is well documented, for example, in political polls or diagnostic questionnaires, where the order of questions can significantly alter responses.

Classical decision models assume that order should not affect the outcome, but empirical data show the opposite, a dynamic that quantum formalism can describe more accurately [66].

Finally, the concept of mental state collapse represents the moment when, from a state of uncertainty or ambivalence, a definite choice is made. This moment is not necessarily the result of rational calculation, but can be sudden, context-sensitive, and influenced by affective, relational, or bodily elements. Collapse does not imply a loss of information, but a crystallization of possibility into a decision-making act: the mind temporarily stabilizes in a meaningful configuration. This aspect is particularly interesting for understanding improvisation, intuition, or creative processes—situations in which action does not follow deductive logic but emerges from a process of emerging configuration.

‘Collapse’ can be conceptualized as the crossing of an affective or cognitive threshold that determines a choice, similar to a decision-making tipping point.

Taken together, these concepts provide a theoretical framework that values the complexity, fluidity, and contextual sensitivity of the human mind. They suggest a view of cognition no longer as a linear sequence of computational operations, but as a dynamic, probabilistic process capable of supporting ambiguity in a functional way.

It is possible, on a theoretical level, to correlate the principles of QC with psychological and psychopathological states and recognize their clinically relevant application (Table 1). This theoretical model appears well-founded and needs to be scientifically validated.

#### The Main Quantum Concepts Applied to Cognition

It is necessary to explain some fundamental concepts of the quantum model applied to the study of decision-making. This explanation will not contain mathematical formalisms but will focus on translating them into intuitive concepts so as not to interrupt the flow of a complex and comprehensive discourse that serves as an introduction to future in-depth studies. In subsequent studies, focused on specific elements of the quantum model applied to psychic processes, the mathematical formulation of the model can be proposed in a limited manner and, therefore, complete and exhaustive for specific needs.

### 3.2. Hilbert Space and Cognitive States

In QC, Hilbert space is a formal metaphor to describe all the possible mental configurations of a subject with respect to a certain content (a decision, a belief, a preference). We can imagine it as a “multidimensional mental space” in which every possible thought, intention, or emotion is represented by a direction. A mental state is a combination of these directions, i.e., a potential configuration of the mind, which can contain multiple alternatives simultaneously. Unlike classical logic, where one thinks of one option at a time, here the mind can “host” multiple possibilities simultaneously, which determines ambivalence, uncertainty, or decision complexity. Every possible thought or intention is a vector in this space, while a mental state is a potential configuration in which multiple vectors coexist simultaneously. This multidimensional representation accounts for the complexity, ambivalence, and uncertainty present in decision-making processes [66,67].

More specifically, each vector in Hilbert space can be associated with an observable psychological dimension, which contributes to the configuration of the mental state. The mental state of a subject is represented by the integration of vectors in multidimensional space.

The lengths of these vectors correspond to the intensity of psychological traits, while their orientation, relative to the axes of Hilbert space, reflects the relative probability of each trait expressing itself in a given context.

In a person with high affective impulsivity, the vectors of mental state will be aligned with the axes representing “impulsivity” and “emotional reactivity,” rather than with the axes representing ‘reflectiveness’ or “tolerance of ambivalence.” This makes the subject more susceptible to quickly collapsing into drastic choices in contexts that call for quick decisions. In an individual with high metacognitive ability and good affective regulation, the vectors will be less oriented toward a single dominant direction, and the person will be able to remain longer in a state of intentional overlap, tolerating ambivalence, suspending decision-making, and evaluating complex alternatives even in contexts that exert strong emotional pressure.

This description allows us to move beyond a static view of psychological traits and represent them as vector components that coexist to varying degrees and interact in determining the overall mental state.

### 3.3. Probabilities as Amplitudes Squared

In the classical decision model, the probability of choosing A or B is simply a number between 0 and 1 that measures how likely A or B is. In QC, however, probability arises from something more complex: the intensity of mind orientation toward a certain option.

This orientation is represented by a “vector” in cognitive space. But the observed probability is proportional to its intensity, which means its “amplitude squared”. In intuitive terms, it is not enough to know that I am thinking about a certain possibility: what matters is how strongly I am thinking about it. It is this intensity (not the mere presence) that determines the probability of the choice. This explains why a choice can emerge suddenly when the intensity exceeds a certain threshold. The probability of that option emerging is therefore proportional to the intensity squared, a concept that explains dynamics such as the sudden appearance of a decision or intuition.

### 3.4. Projection and Collapse of the State

When a decision is made, or an answer is given, the mind “chooses” a trajectory: among the various possibilities contained in the superimposed state, one is realized. This process is called state collapse; in it, the cognitive configuration, which was previously fluid and potential, stabilizes into a single option.

Projection represents the transition from a complex and open mental state to a more defined and “measurable” one, such as when we verbalize a decision or take action. This does not imply that the other options are lost forever: they may reemerge if conditions or context change. But at that moment, the mind “contracts” onto a defined configuration. When we decide or respond, the multifaceted mental state organizes itself around a well-defined option.

Projection is the transition from a potential, multidimensional state to a stable, observable configuration when the mind crystallizes into a concrete option. This does not eliminate the others, which remain in the potential memory, ready to reemerge if the context changes.

### 3.5. Effect of Order: Non-Commutativity

In classical logic, the order in which you ask questions or evaluate options should not change the outcome. But in many real-life cases, such as surveys, clinical interviews, or emotionally relevant decision-making processes, order matters. In QC, this is called non-commutativity: asking first “do you feel guilty?” and then “are you angry?” can lead to a different answer than the reverse order because each question “prepares” the next mental state, directing attention and modifying the configuration of Hilbert’s cognitive space.

The mind is sensitive to the path it is led down, not just the content of the answers. In QC, the order in which questions or information are presented can change the final decision; the decision depends not only on the content, but also on the path through which mental states evolve.

### 3.6. Interference

In cognitive terms, interference means that the way we think about one option can be influenced by other options, even if we do not explicitly choose them. For example, simply considering two alternatives can change the perception of a third. This often occurs when we are undecided: the options “compete” with each other, influencing each other even in the absence of a logical comparison. Interference can explain why preferences change even without new information, and why the presence of multiple alternatives can often confuse rather than clarify. In dynamic terms, it is as if the waves of meaning associated with each option overlap, producing constructive or destructive effects on the decision-making process. Interference describes how cognitive options interact with each other even in the absence of explicit comparison. Thinking about different possibilities simultaneously can generate constructive or destructive effects, which in turn can change the perception or preference for a choice. This mechanism explains phenomena that are problematic for classical theory, such as the disjunction fallacy or preference fluctuation. The quantum model accounts for these phenomena through the overlap and the interaction between “mental waves”.

## 4. Discussion

Personality as a dynamic structure in Hilber’s cognitive space: a quantum application to decision-making under emotional pressure.

From a phenomenological point of view, personality is not conceived as a rigid and stable entity, but as a dynamic structure of pre-reflective dispositions, patterns of embodied meanings, and styles of contact with the world. In this perspective, consistent with phenomenological psychopathology [68,69], personality represents a potential field from which intentionality, emotions, and decision-making tendencies emerge. This field can be formalized as a cognitive Hilbert space, that is, a multidimensional potential space, in which each direction represents a possible experiential and behavioral configuration of the individual, modulated by temperamental traits, affective factors, and deep narrative configurations.

Subjects with fragile or disorganized personality structures (as found in borderline, avoidant, or narcissistic disorders) tend to generate highly unstable overlapping cognitive states, in which divergent decision-making options coexist—in ways that are often irreconcilable and logically unsolvable. In such cases, emotional pressure, generated by internal factors (such as shame, abandonment, fragmentation of identity) or external factors (such as relational conflict, symbolic or concrete threats), acts as a disruptor that forces a premature collapse of the overlapping state toward impulsive, disorganized, or dysfunctional choices [60,66].

For example, patients with borderline personality disorder may find themselves in a state of cognitive overlap when considering whether to maintain or end a significant emotional relationship. In this mental state, opposing intentions (closeness and withdrawal) coexist, yet remain undefined. The emotional pressure resulting from feelings of real or imagined abandonment can act as an affective disturbance, inducing impulsive decision-making collapse, which manifests clinically as relational acting out, sudden emotional breakdown, or intense and destabilizing impulsive reactions.

It is possible to simulate the decision-making behavior of borderline individuals using a computational model inspired by Quantum Cognition. This simulation can be constructed in a Hilbert-space vector environment with the following properties:

- Antithetical behavioral expressions (e.g., “impulsive act” vs. “waiting for events”) are represented by orthogonal axes.
- Affective pressure is modeled as the length of the vector representing a mental state, which makes a collapse in a specific direction more likely.
- Collapse is simulated by projecting the vectors onto the axes representing behavioral expressions. The more unstable the subject is, the more their vectors will be oriented toward impulsive behavioral expressions.

The simulation allows to generate of probability curves of decision collapse based on the intensity of the emotional context (exogenous modulation) and the integrity of the personality structure (endogenous modulation). This strategy makes it possible to produce testable hypotheses, create simulated clinical scenarios, and construct quantitative predictions about decision-making behavior under stress in patients with borderline functioning.

To experimentally verify this dynamic, a controlled clinical study can be structured in three phases:

Phase 1—Induction of intentional overlap.

Participants (affected by BPD) are exposed to ambiguous relational decision-making tasks (e.g., simulated vignettes or ambivalent virtual relational environments). Perceived ambivalence, average choice latency time, and response variability in repeated blocks of the same stimulus are evaluated.

Phase 2—Induction of emotional pressure through disruptive stimuli (emotionally negative feedback or threat of social exclusion). The following are observed: decision-making collapse (abandonment of ambivalence, impulsive choice), neurophysiological changes (HRV, skin conductance, EEG); impact on the sense of post-task narrative coherence.

Phase 3—Psychophysiological and qualitative analysis. Behavioral data are integrated with physiological indices (HRV, fNIRS, or HEG) and with qualitative analysis of the experience.

QC allows us to model these processes by considering certain fundamental properties of affective subjectivity: the overlap of contradictory intentions, the non-commutativity between emotion and reasoning (the way in which an affect retroactively modifies the cognitive configuration), and the interference between options that dissolve or reinforce each other based on the intersubjective context.

In this logic, personality functions as a basis of cognitive states that determines the availability or accessibility of certain mental configurations over others. An individual with high affective instability and poor reflective function, for example, will have a more “compressed” cognitive space, with decision-making trajectories more prone to impulsive collapse. Conversely, an integrated and flexible personality structure will have a broader and more coherent cognitive space, capable of maintaining overlapping states for longer and deferring decision-making collapse to allow for a more articulated affective and symbolic evaluation.

From a therapeutic point of view, this formulation suggests that strengthening the personality structure and expanding the subject experiential space can have direct effects on decision-making quality: it is not just a matter of teaching cognitive strategies, but of modulating the subjective quantum field, i.e., the dynamic space in which meanings take shape even before they become conscious representations or manifest actions [68,69,70].

**Figure 1:**
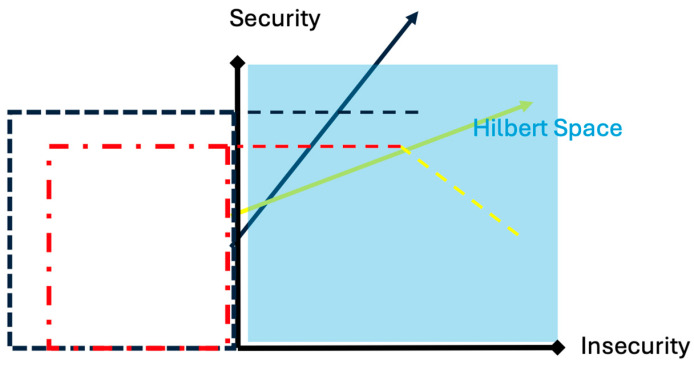
Calculation of the ‘Collapse of Mental State’ before and after the interaction with the environment. In the graph are represented: the Hilbert space (cyan-colored square), the character trait of minimal anxiety of a subject about to take an exam (blue vector in the Hilbert space), the examiner’s markedly anxiety-inducing behavior (yellow vector in the space), and finally, the probability spaces for safe behavior before and after the interaction between examinee and examiner (blue and red squares).

Figure 1 describes, in a simplified geometric form, the calculation of the “collapse of the mental state” before and after the interaction between a character trait of a subject (blue vector) and the relational context (yellow vector). The probability space that the examined subject maintains safe behavior, before the interaction with the examiner, is given by the blue square constructed from the projection of the anxiety vector onto the vertical axis of security. The interaction with an anxiety-inducing examiner leads to a reduction in the probability space that the examinee maintains safe behavior. This non-commutative change in the collapse of the mental state is described by the area of the red square (smaller than the blue square), constructed from the projection of the interaction between the examinee’s anxiety vector and the examiner’s behavior vector.

## 5. Conclusions

The analysis of human decision-making under conditions of uncertainty, conflict, and emotional pressure requires a theoretical framework capable of integrating multiple levels of subjectivity: neurobiological, cognitive, affective, and characterological. Classic models of rationality, even if effective in formal and computational contexts, are often inadequate for capturing the complexity of real-life decision-making experiences, especially when the choice is modulated by ambivalent emotions, tension between motivational plans, and fragile or disorganized personality structures [71]. In this regard, the paradigm of “quantum cognition” offers an innovative conceptual framework capable of dynamically representing the mental configurations that precede and accompany decision-making. The introduction of concepts such as superposition, non-commutativity, interference, and collapse of the cognitive state allows us to model the instability, reversibility, and fluidity inherent in human thought in emotionally charged or highly complex situations.

In particular, the definition of a cognitive Hilbert space allows us to formalize the dynamic coexistence of intentional alternatives, opening up a more faithful representation of subjectivity in action. Personality, understood according to phenomenological and clinical epistemology, cannot be reduced to a sum of traits, but is a dynamic predispositional field in which possibilities of meaning, affective structures, and decision-making trajectories take shape. This field, when viewed through the lens of “quantum cognition” becomes the “probabilistic landscape” on which the subject’s choices unfold: a landscape whose configuration depends not only on content, but also on affective disposition, openness to the world, and the degree of integration of personal identity [72].

This perspective also opens up new possibilities in terms of methodology and modeling. The adoption of formal tools inspired by quantum theory—while devoid of ontological implications about the physical nature of the brain—allows us to represent subjective dynamics that escape classical probability, such as affective ambivalences, the fluctuation of intentional states, and the sudden transformation of meaning. In this framework, the mind no longer appears as a rational computer, but as an open, plastic, and contextual system in which contact with the world retroactively modifies the space of possibilities.

The integration of quantum cognition and phenomenological psychopathology proves particularly fruitful, both for understanding personality disorders and decision-making dysfunctions, and for constructing theoretical models consistent with clinical practice. The experience of choice in subjects with borderline, avoidant, or narcissistic personality organizations, for example, can be interpreted as a premature collapse of the cognitive state, induced by intolerable affective configurations or the fragility of the intentional structure. Similarly, therapeutic work can be seen as a process of expanding the subjective Hilbert space through the reactivation of relational, narrative, and bodily possibilities that were previously excluded or collapsed into rigid and symptomatic patterns. The adoption of formal tools inspired by quantum theory (without ontological implications for the physical structure of the brain) allows for the representation of nonlinear subjective dynamics, such as affective ambivalences, fluctuating intentional states, and sudden transformations of meaning.

As Busemeyer and Bruza [60] write, “the mind, in many conditions, does not follow the classical principles of logic or probability: it exists in a potential state that evolves in context and collapses only at the moment of decision” (p. 4). In this framework, the mind is not an information processor, but a field of possibilities in dialogue with the lived world. The integration of quantum cognition and phenomenological psychopathology appears particularly promising both for understanding decision-making dysfunctions in personality disorders and for outlining therapeutic strategies aimed at expanding the subject’s intentional space.

From this perspective, therapeutic work is not limited to correcting logical distortions, but aims to make the field of possibility habitable again: helping the patient to remain in the overlap, to tolerate ambiguity, to delay decision collapse, until new trajectories of meaning emerge. As Fuchs argues, “therapy does not consist in replacing an incorrect meaning with a correct one, but in opening up a space in which new configurations of meaning can appear” [68] (p. 165).

This view suggests that the future of psychological and clinical research should be based on interdisciplinary and multi-level models capable of combining formal rigor, phenomenological depth, and attention to embodied subjectivity. The contribution of quantum cognition goes in this direction, providing theoretical tools to represent the fluidity of decision-making experience, but also inspiring new therapeutic and diagnostic approaches based on the dynamics of potential mental states and the affective topology of personality.

The challenge for future research will be to construct epistemologically grounded tools that can model the complexity of the mind without reducing it to simplistic schemes, and to produce clinical and psychotherapeutic applications that translate these theoretical frameworks into transformative practices for the subject.

The analysis of personality disorders is currently conducted through two principal methodological approaches. The first relies on a nomothetic and categorical system (DSM–5 TR), in which character dysfunctions are identified based on symptom lists, in which symptoms are marked as either present or absent. The second approach views dysfunctional aspects as dimensional traits, with pathology emerging when a specific trait reaches an intensity that exceeds the threshold of normal variation [73,74].

An approach to personality grounded in quantum equations proposes a view of psychological functioning that is closely linked to the organism–environment field [75].

The quantum approach emerged within the research regarding decision-making as a method for formalizing quantitative and qualitative data; when applied to the study of personality, however, it introduces a distinct epistemological framework to the methodological aspects [76].

At this stage, the Quantum Cognition (QC) model ceases to function solely as an analytical tool and becomes a theoretical framework for understanding the dynamics of personality disorders—one that offers significant diagnostic, methodological, and conceptual potential. This approach—referred to as Quantum Personality—aims to define the probability space within which an individual’s mental state “collapses.” It offers a genuine account of how personality emerges from the interaction between the individual and the environment, as well as the epistemological and psychopathological implications that result from this dynamic.

These issues will be examined more extensively in a forthcoming study, which will present a computational simulation illustrating how psychopathological phenomena arise from the dynamics of the interactive field.

## Figures and Tables

**Table 1:** Fundamental concepts of Quantum Cognition applied to clinical psychopathology.

Quantum Concept	Psychological Definition	Clinical Application
Superposition	Coexistence of incompatible mental states or intentions (e.g., affective ambivalence, indecision).	Tolerate ambivalence in personality disorders.
Interference	Mutual influence between mental options that modifies preference or final outcome.	Explain variability in choices and dysfunctional behaviors.
Order Effect	Effect of the order in which information is presented.	Analyzing the role of therapist-patient interaction in the co-construction of meaning.
Mental state collapse	The moment when a decision is made, often under emotional or cognitive pressure.	Monitor impulsive decision-making as a clinical marker.
Hilbert space	Multidimensional representation of the subject’s potential mental space.	Mapping the personality structure in terms of configurations of possibilities.
Probability amplitude	The intensity with which a possibility is considered increases its probability.	Assessing the preverbal strength of an intention or pattern in subjects with affective dysregulation.

## Data Availability

No new data were created or analyzed in this study. Data sharing is not applicable to this article.

## Abbreviations

The following abbreviations are used in this manuscript:

QC	Quantum Cognition
BPD	Borderline Personality Disorder
EEG	Electroencephalogram
